# Supplementary material for: Fluorescence Cross-Correlation Spectroscopy Yields True Affinity and Binding Kinetics of Plasmodium Lactate Transport Inhibitors
Source: Pharmaceuticals (Basel). 2021 Aug 2;14(8):757. doi: 10.3390/ph14080757 (PMC8399565; doi:10.3390/ph14080757)
Supplement: Supplementary file 1 [file pharmaceuticals-14-00757-s001.zip › pharmaceuticals-1280868-SI.pdf]

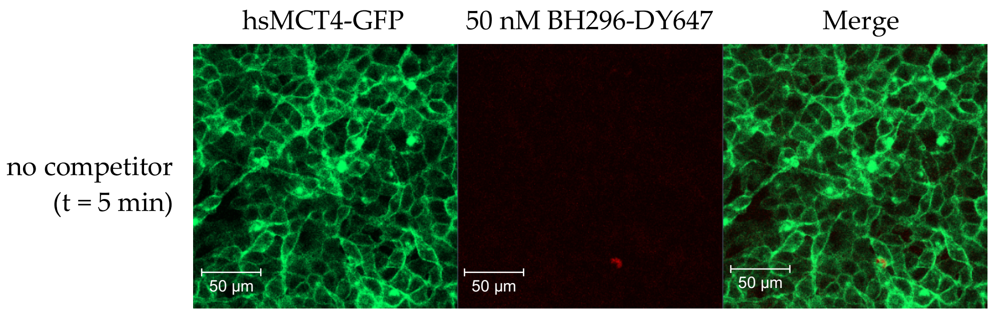


**Figure S1: Specificity of BH296-DY647 for PfFNT** Live cell imaging of hsMCT4-GFP stably expressed in HEK293 (left) cells and coincubation with BH296-DY647 (middle). No co-localization was observed (right).

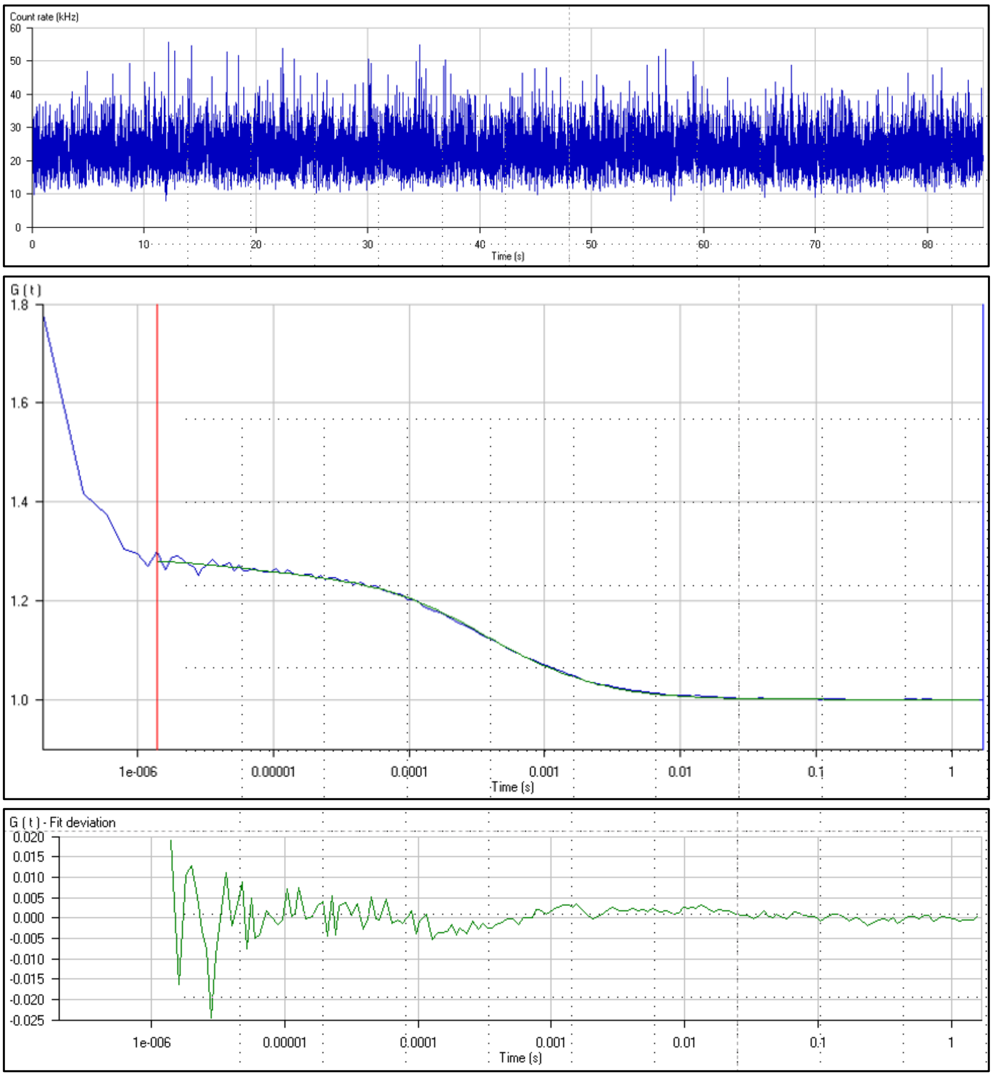


| Channel | Count rate [kHz] | Counts per molecule [kHz] | Amplitude Number particles | Component 1 Diffusion time [µs] |
|---|---|---|---|---|
| GFP | 22.8 | 5.944 | 2.536 | 373 |

**Figure S2: Homogeneous and monodispersed solubilization of PfFNT-GFP** FCS signal fluctuations induced by PfFNT-GFP solubilized in 1% LMNG (upper panel), corresponding correlation curve fitted to a 1 component diffusion model with fit deviation (lower panel).
